# The Gut–Brain–Skin Axis: Systemic Effects of Functional Ingredients in Healthy Skin Aging

**DOI:** 10.3390/ijms27156814

**Published:** 2026-07-29

**Authors:** Yeojin Kim, Sung-Joon Lee

**Affiliations:** 1 Division of Biotechnology, College of Life Sciences and Biotechnology, Korea University, Seoul 02855, Republic of Korea; 2 Department of Food Bioscience and Technology, College of Life Sciences and Biotechnology, Korea University, Seoul 02855, Republic of Korea; 3 Interdisciplinary Program in Precision Public Health, BK21 Four Institute of Precision Public Health, Korea University, Seoul 02846, Republic of Korea

**Keywords:** gut–brain–skin axis, microbiota, skin aging, neuroimmune signaling, functional ingredients, inflammation

## Abstract

Interactions among the gut, brain, and skin are increasingly understood as a systemic regulatory network connecting intestinal activity, neuroimmune communication, and cutaneous homeostasis. Microbiota-derived metabolites, immune mediators, and neuroendocrine pathways can influence central nervous system activity and subsequently regulate skin homeostasis. This review summarizes current evidence on how functional ingredients modulate the gut–brain–skin axis in the context of aging and skin health. Polyphenols, probiotics, and omega-3 fatty acids appear to act through overlapping biological routes, including reshaping microbial communities, supporting epithelial barrier function, regulating immune activity, and limiting oxidative stress. These gut-derived signals may affect the brain through neural, endocrine, and immune pathways, thereby modulating neuroinflammation, hypothalamic–pituitary–adrenal (HPA) axis activity, and neurotransmitter balance. Through brain–skin communication, these changes may influence inflammation, epidermal barrier integrity, collagen remodeling, and skin aging processes. Emerging clinical evidence suggests potential improvements in skin-related outcomes and systemic inflammatory markers; however, studies remain heterogeneous, and integrated assessments of gut, brain, and skin endpoints are limited. Further studies that integrate multi-omics profiling with carefully designed clinical trials will be required to define causal pathways and support the development of evidence-based nutritional approaches targeting this axis.

## 1. Introduction

Increasing life expectancy has intensified the need for strategies that not only extend lifespan but also improve healthspan. Aging involves gradual deterioration across multiple physiological systems and is accompanied by functional changes in both the nervous system and the skin. Cognitive decline, including memory loss and reduced executive function, represents a major burden in aging populations, while skin aging—manifested by wrinkles, loss of elasticity, and impaired barrier function—significantly affects quality of life and psychosocial well-being. Notably, age-related changes in cognition and skin condition are influenced by overlapping systemic processes, such as persistent low-grade inflammation, oxidative imbalance, altered gut microbial ecology, disrupted immune control, and stress-related endocrine signaling. These shared mechanisms suggest that aging-related changes in brain and skin function may reflect broader systemic dysregulation rather than isolated organ-specific processes.

Conventional anti-aging strategies, such as topical cosmetics and aesthetic procedures, primarily target local skin manifestations and visible cutaneous symptoms. Although these approaches can provide cosmetic improvement, they may have limited capacity to address systemic mechanisms underlying aging-related skin dysfunction. In contrast, nutritional interventions using functional ingredients have gained increasing attention because they may modulate aging-related pathways at the systemic level through interactions involving the gut microbiota, immune system, and neuroendocrine signaling.

Growing evidence from nutrition and molecular biology has expanded interest in functional ingredients as modulators of biological pathways involved in aging. Bioactive compounds such as polyphenols, probiotics, peptides, and omega-3 fatty acids have been extensively studied for their antioxidant, anti-inflammatory, and neuroprotective properties. Notably, the biological effects of these compounds cannot always be fully explained by their direct accumulation in skin tissues, given their relatively limited bioavailability following oral intake. Instead, growing evidence suggests that these compounds may exert systemic regulatory effects through microbiota-derived metabolites, neuroimmune signaling, and adaptive stress-response pathways consistent with the concept of hormesis. Beyond their individual effects, these compounds are increasingly recognized for their ability to exert systemic influences through interconnected biological pathways.

Within this perspective, the gut–brain–skin axis offers a useful model for explaining how intestinal, neural, and cutaneous systems interact during aging [1,2,3]. The gut microbiota plays a central role in this axis by regulating immune responses, producing metabolites, and influencing neurotransmitter systems. Dysbiosis has been associated with chronic inflammation, oxidative stress, and disruptions in both brain and skin homeostasis, suggesting that targeting the gut microbiota may provide a unified strategy for addressing multiple aspects of aging [1,2,3].

Despite growing research in this field, many studies remain focused on isolated systems or specific outcomes, with limited integration across organs. In addition, the relatively low bioavailability of many dietary bioactive compounds suggests that direct delivery to skin tissues alone may not sufficiently explain their reported physiological effects. This highlights the need for new mechanistic frameworks capable of explaining how nutritional interventions exert systemic and indirect influences across multiple organs. A more comprehensive understanding of how functional ingredients modulate the gut–brain–skin axis is therefore needed to better define their therapeutic potential.

This axis-based perspective helps explain how microbial and immune signals originating in the gut may affect brain-related pathways and, in turn, influence skin physiology. This interconnected system provides a basis for understanding how microbial-derived signals influence both brain function and skin health, particularly in the context of aging and chronic inflammation. In this review, we provide an integrated overview of key functional ingredients targeting the gut–brain–skin axis, with a focus on their roles in aging, cognitive function, and skin health. We examine underlying biological mechanisms, summarize current preclinical and clinical evidence, and discuss future directions for the development of targeted nutritional strategies. To better understand these interactions, the Section 2 outlines the key biological mechanisms underlying communication within the gut–brain–skin axis.

## 2. The Gut–Brain–Skin Axis: Biological Basis

**Figure 1:**
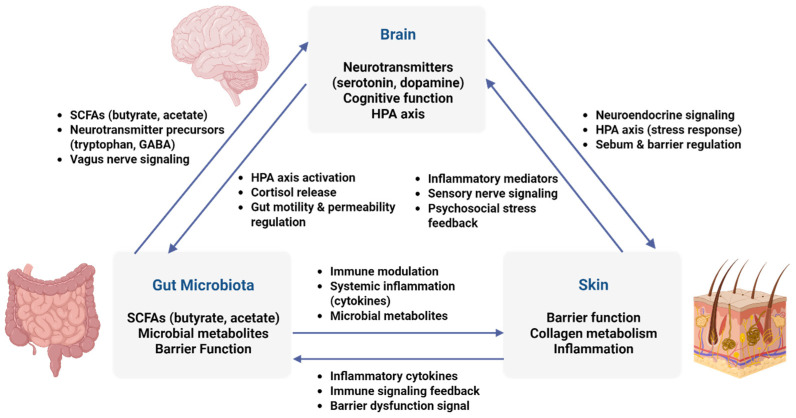
Schematic representation of the gut–brain–skin axis. Diagram summarizing reciprocal communication among the gut microbiota, central nervous system, and skin. Microbial products, such as short-chain fatty acids (SCFAs) and neurotransmitter-related precursors, can affect brain activity through neural, immune-mediated, and endocrine routes. In turn, brain-derived signals, particularly through the hypothalamic–pituitary–adrenal (HPA) axis, regulate gut physiology, including motility, permeability, and microbial composition. The gut–skin axis is mediated by immune modulation, systemic inflammation, and microbial metabolites, while skin-derived inflammatory mediators can feedback to influence gut homeostasis. Similarly, the brain–skin axis operates through neuroendocrine signaling and stress responses, affecting skin barrier function and inflammation. These bidirectional interactions highlight the systemic integration of the gut–brain–skin axis in maintaining physiological homeostasis. Created in BioRender. Lee, S. (2026) https://BioRender.com/5dms92r (accessed on 23 June 2026).

Building on this concept, the gut–brain–skin axis can be viewed as a two-way communication system among the intestine, the central nervous system, and the skin [1,2]. This system is mediated by interactions among the gut microbiota, immune responses, endocrine signaling, and neural pathways, collectively contributing to systemic homeostasis. The bidirectional and integrative nature of these interactions is schematically illustrated in Figure 1.

The microbiota–gut–brain axis represents a well-established pathway through which intestinal microorganisms influence brain function and behavior [1]. Gut microbiota produce bioactive metabolites, including short-chain fatty acids (SCFAs), neurotransmitters (e.g., γ-aminobutyric acid [GABA] and serotonin), and microbial-derived peptides. These molecules modulate neural signaling via the vagus nerve, systemic circulation, and immune pathways, thereby influencing neuroinflammation, blood–brain barrier integrity, and neurotransmitter balance [1]. Notably, gut-derived metabolites such as SCFAs and neurotransmitters can enter systemic circulation or signal via the vagus nerve, thereby modulating neuroinflammation and hypothalamic–pituitary–adrenal (HPA) axis activity; these brain-mediated changes subsequently influence skin physiology by altering immune responses, barrier function, and inflammatory signaling [4,5,6,7].

In parallel, the gut–skin axis describes the influence of intestinal microbiota on skin physiology [2,3]. Dysbiosis has been associated with increased intestinal permeability (“leaky gut”), allowing pro-inflammatory molecules such as lipopolysaccharides (LPS) to enter systemic circulation. This process promotes systemic inflammation, which can impair skin barrier function, alter sebum production, and contribute to inflammatory skin conditions [2,3]. Importantly, LPS-driven systemic inflammation can also affect brain function by promoting neuroinflammatory responses, thereby further amplifying downstream effects on skin homeostasis through neuroimmune pathways [4,6,8].

The immune system serves as a central link between these axes. Gut microbiota shape both innate and adaptive immunity, and microbial imbalance can lead to chronic low-grade inflammation characterized by elevated cytokines such as interleukin-6 (IL-6) and tumor necrosis factor-α (TNF-α). These inflammatory mediators can circulate systemically and influence both brain function—by altering neuroinflammatory signaling and synaptic plasticity—and skin physiology by impairing barrier integrity and promoting inflammatory responses [4,6,9].

Neuroendocrine signaling further integrates this system. The HPA axis is a central neuroendocrine stress-response system that regulates cortisol production and systemic physiological adaptation to stress. Stress-induced cortisol release can disrupt gut microbiota composition [1], impair skin barrier integrity [2,3], and alter immune activity [4,6,9]. Conversely, microbial metabolites, particularly SCFAs and tryptophan-derived metabolites, can modulate HPA axis activity by influencing vagal signaling, neuroinflammatory pathways, and neurotransmitter systems involved in stress regulation. These interactions highlight the bidirectional nature of this regulatory network [1]. Through this pathway, gut microbiota–driven alterations in stress signaling can indirectly affect skin health by modulating systemic inflammation, immune responses, and barrier function via brain-mediated endocrine pathways [4,6,10].

Together, these pathways highlight coordinated cross-organ signaling among the gut, brain, and skin. Importantly, these interactions are not isolated but form an integrated network in which gut-derived signals influence brain function, and brain-mediated responses subsequently shape skin physiology, reinforcing the concept of a unified and functionally integrated gut–brain–skin axis [2,3]. This integrative framework provides a basis for understanding how age-related dysregulation affects multiple organ systems simultaneously, as discussed in the Section 3.

In addition to gut-derived microbial signals, the local cutaneous microbiome should be considered as an important component of the gut–brain–skin axis. Aging-related changes in the skin microenvironment, including altered pH, lipid composition, hydration, immune tone, and barrier integrity, can reshape cutaneous microbial ecology [11]. Loss of microbial balance at the skin surface may weaken barrier function, promote localized inflammation, and increase susceptibility to external stressors. Thus, healthy skin aging is unlikely to be determined solely by gut-derived systemic signals, but rather by coordinated interactions among intestinal microbial metabolites, systemic immune regulation, neuroendocrine signaling, and local skin microbiota–barrier homeostasis. This perspective also suggests that bidirectional communication may occur between the gut and skin microbiomes, whereby intestinal dysbiosis influences skin immunity and barrier function, while persistent skin inflammation may feed back into systemic immune regulation.

## 3. Aging-Related Dysregulation of the Gut–Brain–Skin Axis

During aging, progressive functional decline is accompanied by interconnected biological alterations, including oxidative imbalance, sustained low-grade inflammation, mitochondrial impairment, and changes in microbiome composition. In the gut–brain–skin axis, these hallmarks are reflected by increased reactive oxygen species (ROS), elevated pro-inflammatory mediators such as IL-6, TNF-α, and LPS, altered HPA axis activity, and reduced production of microbiota-derived metabolites such as SCFAs. These processes are closely interconnected and contribute to dysregulation of the gut–brain–skin axis [1,3,4,5,6,10].

Oxidative stress plays a central role in aging by promoting cellular damage through the accumulation of ROS. Elevated ROS levels impair neuronal function, accelerate skin aging through collagen degradation, and disrupt gut epithelial integrity. Importantly, oxidative stress does not occur in isolation within a single organ but can develop simultaneously across multiple tissues during aging. Increased ROS production has been observed in the intestinal epithelium, central nervous system, and skin, where cumulative oxidative damage contributes to barrier dysfunction, mitochondrial impairment, neuroinflammation, and extracellular matrix degradation. Moreover, oxidative stress originating in the gut can alter microbiota composition and barrier function, leading to the systemic release of pro-oxidative and pro-inflammatory mediators that influence brain function and subsequently exacerbate skin aging through neuroimmune and inflammatory pathways [4,5,6,12]. In parallel, oxidative stress generated in the brain may further amplify systemic inflammatory signaling and neuroendocrine dysregulation, thereby contributing to impaired skin homeostasis and reinforcing a bidirectional gut–brain–skin pathogenic loop.

Persistent low-grade inflammation, commonly described as “inflammaging,” also contributes substantially to age-associated axis disruption. Increased levels of pro-inflammatory cytokines impair synaptic plasticity in the brain, weaken skin barrier function, and exacerbate gut permeability. Notably, gut-derived inflammatory mediators can enter systemic circulation and modulate neuroinflammation, which in turn affects skin physiology by promoting inflammatory responses and impairing barrier integrity, thereby establishing a gut–brain–skin inflammatory loop [4,6,12,13]. Persistent inflammatory signaling can further aggravate oxidative stress and mitochondrial dysfunction across the axis, accelerating age-associated deterioration in both neurological and skin-related functions.

Mitochondrial dysfunction further contributes to axis dysregulation by reducing cellular energy production and increasing oxidative stress. In neurons, this affects cognitive performance and promotes neurodegeneration. In skin cells, it accelerates aging-related changes such as wrinkle formation and loss of elasticity. In the gut, mitochondrial impairment can weaken epithelial barrier function and disrupt host–microbe interactions. These alterations can propagate across the gut–brain–skin axis, as impaired gut metabolism influences systemic energy balance and stress-related signaling, ultimately affecting skin homeostasis through HPA axis dysregulation and neuroimmune pathways.

Microbiome imbalance (dysbiosis), which becomes more pronounced with age, represents another critical factor. Reduced microbial diversity and loss of beneficial bacteria, including *Bifidobacterium* and *Lactobacillus* species, together with expansion of opportunistic or pro-inflammatory microbial populations, can lead to decreased production of metabolites such as SCFAs. This shift impairs immune regulation, increases gut permeability, and disrupts signaling along the axis [3]. Consequently, reduced microbial metabolite production can alter gut–brain communication by affecting neurotransmitter systems and HPA axis activity, which subsequently influences skin health through changes in immune responses and barrier function [4,5,6,10].

Importantly, these hallmarks are not independent but highly interconnected. For example, microbiome imbalance can exacerbate inflammation and oxidative stress, while mitochondrial dysfunction can amplify these effects by further increasing ROS production and impairing cellular repair mechanisms. Similarly, chronic inflammation can alter microbial composition and barrier integrity, thereby promoting additional endotoxin translocation and neuroimmune activation. These processes collectively form a self-reinforcing cycle in which dysbiosis, oxidative stress, mitochondrial dysfunction, and inflammaging continuously interact across the gut, brain, and skin. As a result, disruption of the gut–brain–skin axis emerges as a unifying mechanism underlying age-related decline in both cognitive function and skin health [1,2,3]. This integrated perspective supports the concept that aging-related dysfunction is driven not by isolated organ damage, but by progressive dysregulation of interconnected systemic signaling networks.

Importantly, skin aging associated with the gut–brain–skin axis should be distinguished from localized skin aging processes occurring primarily within cutaneous tissues. Conventional mechanisms of skin aging involve direct ultraviolet (UV)-induced oxidative damage, fibroblast senescence, collagen degradation, matrix metalloproteinase (MMP) activation, and local inflammatory signaling within the skin microenvironment. Accordingly, topical cosmetics, laser-based procedures, and dermatological interventions primarily target local tissue remodeling and visible cutaneous symptoms. However, these approaches may have limited capacity to address the underlying systemic and inflammatory mechanisms contributing to age-associated skin dysfunction.

In contrast, gut–brain–skin axis–mediated skin aging involves systemic dysregulation originating from gut microbiota imbalance, chronic low-grade inflammation, neuroendocrine stress responses, and immune dysfunction. These systemic alterations can indirectly influence skin physiology through circulating inflammatory mediators, oxidative stress, HPA axis dysregulation, and neuroimmune signaling pathways. Therefore, modulation of the axis through dietary and functional ingredient-based approaches may provide broader systemic benefits by targeting upstream mechanisms underlying chronic inflammation and age-associated physiological decline. This distinction highlights the complementary relationship between conventional skin-directed interventions and systemic axis-based strategies for promoting healthy skin aging.

Although systemic inflammation is a shared feature of both aging and inflammatory skin diseases, its biological meaning and clinical consequences are not identical. In healthy skin aging, inflammation is typically characterized by chronic, low-grade systemic inflammatory tone, often described as inflammaging, accompanied by oxidative stress, mitochondrial dysfunction, extracellular matrix remodeling, and gradual impairment of barrier function [14]. In contrast, common inflammatory skin diseases such as atopic dermatitis and psoriasis involve more disease-specific immune pathways, including type 2 immune responses in atopic dermatitis and IL-23/Th17-related inflammation in psoriasis [15,16]. Therefore, evidence derived from inflammatory skin diseases should not be interpreted as direct proof of efficacy in healthy skin aging. Rather, such evidence provides indirect translational support for the concept that modulation of gut dysbiosis, intestinal permeability, systemic inflammation, and immune signaling may influence skin outcomes.

This distinction is clinically important because many interventions discussed in this review, including probiotics and omega-3 fatty acids, have been more extensively evaluated in inflammatory skin diseases than in aging-specific skin endpoints. Outcomes such as reductions in erythema, lesion severity, pruritus, or atopic dermatitis scores reflect inflammatory disease activity, whereas outcomes such as wrinkle depth, elasticity, hydration, collagen degradation, transepidermal water loss, and photoaging markers more directly represent skin aging. Future studies should therefore separate aging-specific outcomes from inflammatory disease outcomes and clarify whether axis-targeted interventions prevent age-associated skin decline, reduce inflammatory disease activity, or affect both processes through overlapping but distinct mechanisms. These differences in inflammatory patterns, clinical outcomes, and evidence interpretation are summarized in Table 1.

**Figure 2:**
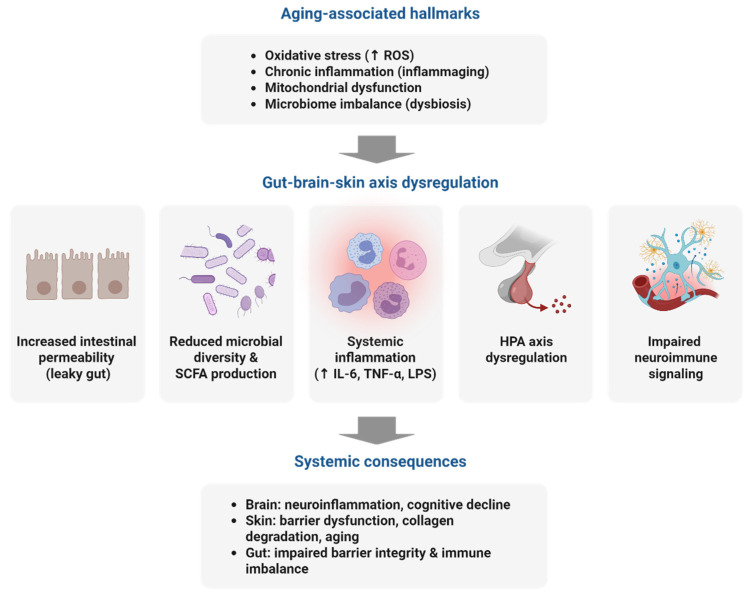
Aging-related disruption of gut–brain–skin communication. Diagram illustrating how aging-associated biological changes disturb signaling across the gut–brain–skin axis. Major features include oxidative stress, persistent inflammation (“inflammaging”), mitochondrial impairment, and microbiome imbalance. These changes contribute to impaired gut barrier integrity, reduced microbial diversity, and increased intestinal permeability, facilitating the systemic dissemination of pro-inflammatory mediators. Such systemic alterations promote neuroinflammation, dysregulation of the HPA axis, and impaired neurotransmitter balance in the brain. Concurrently, these processes negatively affect skin physiology by weakening barrier function, increasing oxidative damage, and accelerating collagen degradation. Together, these interconnected alterations illustrate how aging drives coordinated dysfunction across the gut, brain, and skin, ultimately contributing to cognitive decline and skin aging. Created in BioRender. Lee, S. (2026) https://BioRender.com/0z4ouh3 (accessed on 23 June 2026).

Together, these observations and the distinctions summarized in Table 1 suggest that aging is a systemic process driven by network-level dysregulation rather than isolated organ dysfunction [1]. These systemic alterations provide a biological basis for targeting the gut–brain–skin axis using functional compounds. Accordingly, interventions that modulate these interconnected pathways may represent a promising strategy for restoring axis homeostasis, as discussed in the following sections. The aging-associated mechanisms contributing to gut–brain–skin axis dysregulation are summarized in Figure 2.

## 4. Key Food-Derived Functional Ingredients Modulating the Gut–Brain–Skin Axis

These ingredients appear to influence several common biological targets, including microbial composition, epithelial barrier function, immune balance, and neuroendocrine routes involving vagal signaling and the HPA axis [5,7,10,17]. Through these interconnected processes, gut-derived metabolites and immune signals may influence neuroimmune and neuroendocrine pathways in the brain, which subsequently affect skin physiology and systemic homeostasis [1,2,3,6,10,11]. This perspective suggests that the beneficial effects of orally administered functional ingredients on skin health may arise not only from direct tissue exposure, but also from indirect systemic regulation mediated through the gut–brain–skin axis [2,3,5,11].

### 4.1. Polyphenols

Polyphenols represent one of the most extensively investigated classes of bioactive compounds influencing the gut–brain–skin axis. Consumption of polyphenol-rich foods and supplements has been linked to antioxidant, anti-inflammatory, and barrier-supporting effects relevant to skin health and aging [13]. However, the relatively low bioavailability of many polyphenols in skin tissues suggests that their beneficial effects may not be explained solely by direct accumulation within the skin. Rather, available evidence indicates that polyphenols may act systemically by influencing the gut–brain–skin axis, including gut microbiota composition, intestinal barrier function, and inflammatory or oxidative signaling pathways [4,5,6,8,9,13,17]. Through these interconnected mechanisms, polyphenols enable coordinated communication between the gut, brain, and skin, ultimately contributing to systemic homeostasis and skin health.

#### 4.1.1. Flavonols—Quercetin

Quercetin is a plant-derived flavonol found in many fruits and vegetables and has been investigated for anti-inflammatory, antioxidant, and barrier-supporting effects [13,18,19]. Recent studies suggest that quercetin may contribute to gut–brain–skin axis regulation through microbiota-dependent and host-mediated mechanisms.

In intestinal experimental systems, both cell-based and animal studies indicate that quercetin can alter microbial composition by increasing beneficial taxa, including *Bifidobacterium* and *Lactobacillus*, while reducing opportunistic pathogens [20,21,22,23]. This shift is accompanied by increased production of SCFAs, which are critical for maintaining epithelial integrity and immune balance [5]. Experimental animal studies further indicate that quercetin supports intestinal barrier function by increasing tight junction proteins such as zonula occludens-1 (ZO-1), occludin, and claudins, thereby reducing intestinal permeability and limiting systemic dissemination of endotoxins such as LPS [24,25,26].

Gut-derived alterations are also linked to reductions in systemic inflammation and neuroinflammatory responses. In the brain, in vitro mechanistic studies indicate that quercetin reduces inflammatory signaling by suppressing nuclear factor kappa B (NF-κB) activation and downstream cytokines, including TNF-α and IL-6 [27,28,29]. In parallel, stimulation of antioxidant defense pathways via nuclear factor erythroid 2–related factor 2 (Nrf2) contributes to reduced oxidative stress and neuronal protection [30,31]. In vivo studies have additionally demonstrated reduced microglial activation and attenuation of neuroinflammatory responses, suggesting a modulatory role in neuroimmune interactions [12].

Regarding cutaneous effects, quercetin exerts protective actions by reducing oxidative stress and inflammatory responses. Cell-based and animal studies have reported that quercetin inhibits ROS production and suppresses the expression of matrix metalloproteinases (MMPs), thereby preventing collagen degradation and supporting dermal structure [32,33,34]. These effects contribute to improved skin barrier function and attenuation of aging-related processes [35,36]. Although the current evidence is largely preclinical, early human studies suggest improvements in selected skin parameters and inflammatory markers following quercetin-rich dietary or supplemental interventions, supporting its potential relevance to microbiota-, neuroimmune-, and skin-related outcomes [37,38].

Collectively, these observations indicate that quercetin-mediated changes in gut microbiota composition and SCFA production may strengthen intestinal barrier integrity and reduce systemic translocation of endotoxins such as LPS [5,20,21,22,24,25,26]. This reduction in peripheral inflammatory signaling may then affect brain-related immune pathways by suppressing microglial activation, NF-κB signaling, and pro-inflammatory cytokine production while enhancing Nrf2-mediated antioxidant responses [12,27,28,29,30,31]. Through regulation of neuroimmune and neuroendocrine signaling, these brain-mediated effects may contribute to reduced cutaneous inflammation, preservation of collagen homeostasis, and improved skin barrier function [32,33,34,35,36,37,38]. Therefore, the dermatological benefits of orally administered quercetin may arise not only from direct antioxidant activity within skin tissues but also from coordinated systemic regulation along the gut–brain–skin axis [5,12,20,21,22,24,25,26,27,28,29,30,31,32,33,34,35,36,37,38]. However, because most available studies have examined intestinal, neural, and cutaneous outcomes separately, direct causal evidence demonstrating the complete gut-to-brain-to-skin sequence in a single integrated quercetin study remains limited. Future studies integrating microbiome profiling, microbial metabolite analysis, neuroimmune or neuroendocrine endpoints, and dermatological outcomes will be required to validate this axis-level mechanism.

#### 4.1.2. Flavonols—Epigallocatechin-3-gallate (EGCG)

EGCG is the predominant catechin in green tea and has been characterized for anti-inflammatory and antioxidant activity [39,40,41]. Available evidence indicates that EGCG may influence gut–brain–skin axis regulation by affecting gut microbiota composition, intestinal barrier integrity, neuroinflammatory signaling, and cutaneous photoaging responses.

In the gut, fermentation studies and animal models indicate that EGCG can modulate microbial composition by promoting beneficial bacterial populations, including *Lactobacillus* and *Bifidobacterium* species, while suppressing pathogenic taxa [42,43,44]. Studies using intestinal epithelial cell and murine colitis models further indicate that EGCG supports intestinal barrier integrity by increasing tight junction protein expression, thereby reducing permeability and endotoxin translocation [45,46].

In the brain, microglial and neuronal cell studies indicate that EGCG reduces neuroinflammation by inhibiting NF-κB signaling and decreasing inflammatory cytokine production [47,48,49]. In vivo studies in rodent models further reported reduced microglial activation, attenuation of oxidative stress, and improved neuronal resilience following EGCG administration [30,31].

In the skin, EGCG demonstrates well-established photoprotective and anti-aging effects. In vitro studies using human dermal fibroblasts and keratinocytes have shown that EGCG inhibits UV-induced expression of MMPs and inflammatory mediators while preserving collagen integrity [50,51,52]. Animal studies using UV-induced photoaging models further demonstrated reductions in oxidative damage and wrinkle formation. In human intervention studies, green tea catechin supplementation has been linked to reduced erythema, improved skin structure, and protection against photoaging [53,54,55,56].

Collectively, these observations indicate that EGCG-mediated changes in gut microbiota composition and intestinal barrier integrity may reduce permeability, endotoxin translocation, and peripheral inflammatory signaling [42,43,45,46]. These gut-associated changes may subsequently influence neuroimmune pathways by suppressing NF-κB signaling, reducing inflammatory cytokine production, and attenuating microglial activation and oxidative stress in the brain [44,47,48]. Through these neuroimmune and antioxidant pathways, EGCG may contribute to reduced UV-induced inflammation, suppression of MMP expression, preservation of collagen integrity, and protection against photoaging-related skin damage [50,51,52,53,54,55,56]. Therefore, the dermatological benefits of orally administered EGCG may arise not only from direct antioxidant and photoprotective effects within skin tissues but also from coordinated systemic regulation along the gut–brain–skin axis [42,43,44,45,46,47,48,49,50,51,52,53,54,55,56]. However, because most available studies have evaluated intestinal, neural, and cutaneous outcomes separately, direct causal evidence demonstrating the complete gut-to-brain-to-skin sequence in a single integrated EGCG study remains limited. Future studies integrating microbiome profiling, intestinal permeability markers, neuroinflammatory endpoints, oxidative stress biomarkers, and dermatological outcomes will be needed to validate this axis-level mechanism.

#### 4.1.3. Stilbenes—Resveratrol

Resveratrol is a stilbene compound present in grapes and berries and has been investigated for anti-inflammatory, antioxidant, and metabolic regulatory activities [57,58,59,60]. Available evidence indicates that resveratrol may influence gut–brain–skin axis regulation by affecting microbial composition, intestinal barrier integrity, sirtuin 1 (SIRT1)/AMP-activated protein kinase (AMPK) signaling, HPA axis activity, and cutaneous oxidative stress responses.

In the gut, in vivo studies using high-fat diet and metabolic inflammation animal models have shown that resveratrol reshapes microbial composition and reduces metabolic endotoxemia [61,62,63]. Experimental studies further showed improved intestinal barrier integrity through regulation of tight junction proteins, thereby decreasing LPS translocation and systemic inflammation [24,25,26]. These effects extend to the brain, where neuronal and glial cell studies indicate that resveratrol regulates cellular stress responses through activation of SIRT1 and AMPK signaling pathways [64,65,66]. In vivo studies in aging and neurodegenerative animal models additionally reported modulation of HPA axis activity, reduced neuroinflammation, attenuation of oxidative stress, and protection against neuronal damage [10,58,67,68,69].

In the skin, in vitro studies using dermal fibroblasts have shown that resveratrol promotes collagen synthesis, inhibits MMP activity, and suppresses inflammatory pathways [70,71,72]. Animal studies further showed protection against UV-induced oxidative damage and wrinkle formation, while early clinical studies reported improved skin elasticity and aging-related skin parameters following oral supplementation [73,74,75].

Collectively, these observations indicate that resveratrol-mediated changes in gut microbiota composition and intestinal barrier integrity may reduce metabolic endotoxemia, LPS translocation, and systemic inflammatory signaling [24,25,26,61,62,63]. These gut-associated changes may subsequently interact with brain-related pathways by activating SIRT1/AMPK signaling, modulating HPA axis activity, and attenuating neuroinflammation and oxidative stress [10,58,64,65,66,67,68,69]. Through these neuroendocrine and neuroimmune mechanisms, resveratrol may contribute to preservation of collagen structure, suppression of MMP activity, protection against UV-induced oxidative damage, and improvement of aging-related skin parameters [70,71,72,73,74,75]. Therefore, the dermatological benefits of orally administered resveratrol may arise not only from direct antioxidant activity within skin tissues but also from coordinated systemic regulation along the gut–brain–skin axis [10,24,25,26,58,61,62,63,64,65,66,67,68,69,70,71,72,73,74,75]. However, because most available studies have evaluated intestinal, neural, metabolic, and cutaneous outcomes separately, direct causal evidence demonstrating the complete gut-to-brain-to-skin sequence in a single integrated resveratrol study remains limited. Future studies integrating microbiome profiling, intestinal permeability markers, metabolic endotoxemia, SIRT1/AMPK activity, HPA axis-related endpoints, neuroinflammatory markers, and dermatological outcomes will be needed to validate this axis-level mechanism.

#### 4.1.4. Other Polyphenols

Other polyphenols, including kaempferol, anthocyanins, and curcumin, contribute to the gut–brain–skin axis through overlapping yet less comprehensively characterized mechanisms [76,77,78,79]. These compounds are generally associated with antioxidant and anti-inflammatory activities, changes in gut microbiota composition, and reduced oxidative stress across multiple biological systems. For kaempferol, preclinical studies indicate effects on gut microbiota composition, intestinal inflammation, and intestinal barrier function through tight junction regulation [80,81].

For instance, in vitro, animal, and review-level evidence suggests that anthocyanins can modulate gut microbiota composition and microbial diversity, while anthocyanin-rich extracts have been shown to protect against UV-induced skin damage through antioxidant mechanisms [82,83,84,85]. Curcumin has shown anti-inflammatory effects in macrophage and intestinal epithelial cell models, partly through suppression of Toll-like receptor 4 (TLR4)/NF-κB signaling, improvement of intestinal barrier integrity, and modulation of gut microbiota composition [86,87,88,89,90,91]. Although these compounds exhibit less integrative effects, their ability to modulate microbiota composition and inflammatory signaling suggests a potential indirect contribution to skin health through systemic gut–brain–skin axis regulation rather than solely through localized cutaneous activity [76,77,78,79,86,87,88].

### 4.2. Probiotics

Probiotics influence the gut–brain–skin axis by modulating microbial ecosystems, supporting epithelial barrier function, and regulating immune and neuroactive signaling pathways [92,93,94,95].

#### 4.2.1. *Lactobacillus rhamnosus* GG (LGG)

LGG is one of the most extensively studied probiotic strains, with evidence supporting its effects on intestinal barrier function, immune regulation, and selected clinical outcomes [96,97,98]. Current evidence suggests that LGG may contribute to gut–brain–skin axis regulation by strengthening intestinal barrier integrity, reducing endotoxin-related inflammatory signaling, modulating stress-related neuroimmune pathways, and influencing inflammatory skin outcomes.

In the gut, both cell-based intestinal epithelial studies and animal models have shown that LGG supports barrier integrity by upregulating tight junction proteins and mucin production, thereby reducing intestinal permeability and LPS translocation [99,100,101]. Through the gut–brain axis, LGG influences neuroendocrine and neurotransmitter systems. Experimental studies using *Lactobacillus rhamnosus* strains have reported modulation of gut–brain stress-related signaling, including GABAergic pathways, and reductions in stress-related hormone responses, indicating a role in neuroimmune communication and stress regulation [7,102,103,104]. Regarding cutaneous outcomes, LGG has shown clinically relevant potential, particularly in inflammatory skin conditions such as atopic dermatitis. Randomized controlled trials and clinical investigations in atopic dermatitis have reported potential improvements in disease severity, skin barrier function, and inflammatory markers following LGG supplementation, although outcomes remain strain-, population-, and study design-dependent [105,106,107,108,109,110,111,112,113,114,115].

Collectively, these findings suggest that LGG-mediated enhancement of intestinal barrier integrity may reduce LPS translocation and systemic inflammatory signaling [99,100,101]. These gut-associated changes may subsequently interact with stress-related neuroimmune pathways, including GABAergic signaling, neuroendocrine responses, and immune regulation [7,102,103,104]. Through these systemic and brain-mediated pathways, LGG may contribute to reduced inflammatory skin responses, improved barrier function, and attenuation of atopic dermatitis-related outcomes [105,106,107,108,109,110,111,112,113,114,115]. Therefore, the dermatological benefits of orally administered LGG may arise not only from direct modulation of gut microbiota or local immune responses but also from coordinated systemic regulation along the gut–brain–skin axis [7,99,100,101,102,103,104,105,106,107,108,109,110,111,112,113,114,115]. However, because most studies have evaluated intestinal, neuroimmune, or cutaneous outcomes separately, direct causal evidence demonstrating the complete gut-to-brain-to-skin sequence in a single integrated LGG study remains limited. Future studies integrating gut microbiota profiling, intestinal permeability markers, LPS or inflammatory cytokine measurements, neuroendocrine or stress-related endpoints, and dermatological outcomes will be needed to validate this axis-level mechanism.

#### 4.2.2. Other Probiotic Strains

Other probiotic strains, including *Bifidobacterium longum* and various *Lactobacillus* species, have demonstrated potential in modulating immune responses and improving skin health [92,93,94,95]. These effects are mediated through microbial metabolite production, immune regulation, and stress-related signaling pathways. For example, selected *Bifidobacterium longum* strains have been associated with modulation of stress-related outcomes in human exploratory trials and improvement of skin barrier dysfunction in atopic dermatitis-like models, although these effects remain strain- and context-dependent [116,117,118]. However, probiotic effects are highly strain-specific, and clinical outcomes remain variable. As such, while these strains contribute to the broader understanding of microbiota–host interactions, their roles within the gut–brain–skin axis require further validation.

### 4.3. Docosahexaenoic Acid (DHA) and Eicosapentaenoic Acid (EPA)

Omega-3 fatty acids are key lipid mediators with well-established anti-inflammatory and immunomodulatory properties, contributing to coordinated regulation across the gut–brain–skin axis [119,120,121,122]. DHA and EPA exert multi-level effects through regulation of inflammatory resolution pathways [119,120,121,122]. In the gut, animal and human dietary intervention studies have demonstrated that these fatty acids improve microbial balance and reduce inflammation by modulating immune responses and lipid signaling pathways [123,124,125]. In the brain, neuronal cell studies and animal models have shown that DHA and EPA can serve as precursors to specialized pro-resolving mediators (SPMs), including resolvins and protectins, which actively resolve neuroinflammation and support neuronal function [126,127,128].

Clinical evidence, including randomized controlled trials in inflammatory skin disorders such as atopic dermatitis and psoriasis, indicates that omega-3 supplementation may reduce erythema, improve skin hydration, and enhance barrier repair [129,130,131,132]. Collectively, these observations indicate that DHA and EPA may influence gut–brain–skin axis regulation by modulating gut microbial balance, immune responses, and lipid-derived inflammatory resolution pathways [123,124,125,126,127,128]. In the gut, omega-3 fatty acid–associated changes in microbial composition and immune signaling may reduce peripheral inflammatory tone, thereby providing an upstream mechanism for systemic regulation [123,124,125]. These effects may subsequently interact with brain-related pathways through production of specialized pro-resolving mediators, including resolvins and protectins, which attenuate neuroinflammation and support neuronal homeostasis [126,127,128]. Through these systemic anti-inflammatory and neuroimmune mechanisms, orally administered DHA and EPA may contribute to improved skin barrier function, reduced erythema, enhanced hydration, and attenuation of inflammatory skin disorders, including atopic dermatitis and psoriasis [129,130,131,132]. Therefore, the dermatological benefits of omega-3 fatty acids may arise not only from direct incorporation into skin tissues or local anti-inflammatory effects, but also from coordinated systemic regulation along the gut–brain–skin axis [119,120,121,122,123,124,125,126,127,128,129,130,131,132]. However, because most available studies have evaluated gut microbiota, neuroinflammatory, or dermatological outcomes separately, direct causal evidence demonstrating the complete gut-to-brain-to-skin sequence in a single integrated DHA/EPA study remains limited. Future studies integrating microbiome profiling, lipid mediator analysis, inflammatory cytokine measurements, neuroimmune endpoints, and dermatological outcomes will be needed to validate this axis-level mechanism.

### 4.4. Peptides and Amino Acid–Derived Metabolites

Peptides and amino acid-derived metabolites contribute to the gut–brain–skin axis primarily through supportive and complementary mechanisms [133,134,135].

Collagen peptides enhance dermal structure by promoting extracellular matrix synthesis and improving skin elasticity [133,134,135]. Meanwhile, tryptophan-derived metabolites, including indole compounds, influence gut–brain communication by modulating neurotransmitter pathways and immune responses [136,137,138,139]. Although these compounds demonstrate beneficial effects, their actions are generally more localized or supportive compared to polyphenols, probiotics, and omega-3 fatty acids. Nevertheless, their influence on gut-derived metabolites, microbial signaling, and neuroimmune pathways suggests a potential indirect contribution to skin health through systemic gut–brain–skin axis regulation [136,137,138,139]. Taken together, these findings indicate that diverse functional compounds, such as polyphenols, probiotics, and omega-3 fatty acids, act through interconnected processes across the gut–brain–skin axis. While each compound exhibits distinct molecular targets, a convergence emerges at the level of microbial modulation, barrier integrity, immune regulation, and oxidative stress control [5,12,35,36,92,93,94,95,119,120,121,122].

**Figure 3:**
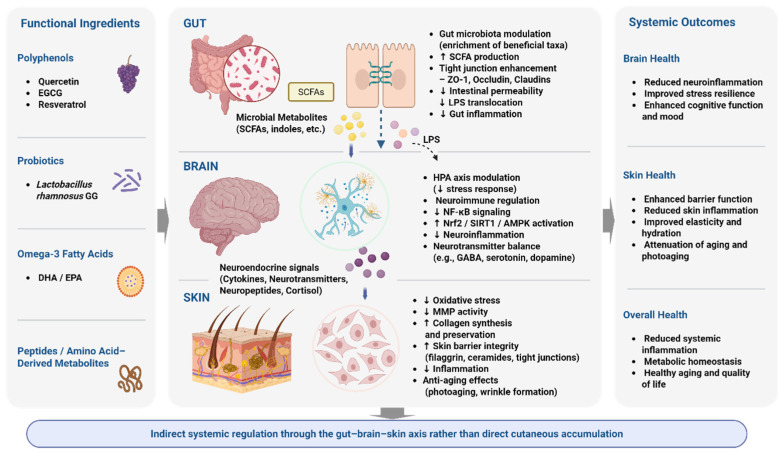
Integrated mechanisms by which functional ingredients influence gut–brain–skin communication and systemic health outcomes. The diagram illustrates how polyphenols, probiotics, omega-3 fatty acids, and amino acid–derived metabolites converge on shared biological processes across the gut–brain–skin axis. These ingredients can alter gut microbiota composition, support intestinal barrier integrity, regulate immune and oxidative stress pathways, and affect neuroendocrine signaling, including HPA axis activity and neuroimmune communication. Gut-derived microbial metabolites and immune mediators subsequently influence brain function and stress-related signaling pathways, which in turn affect skin physiology by regulating inflammation, barrier integrity, collagen homeostasis, and aging-related processes. Collectively, these coordinated interactions support systemic homeostasis and suggest that the beneficial effects of orally administered functional ingredients on skin health may arise through indirect systemic regulation across the gut–brain–skin axis rather than solely through direct accumulation within skin tissues. These relationships are further organized in Table 2 to highlight compound-specific mechanisms and levels of clinical evidence. Created in BioRender. Lee, S. (2026) https://BioRender.com/x49y0i9 (accessed on 23 June 2026).

To better define these shared pathways and their systemic integration, the underlying mechanisms of action are further examined in the Section 5. These integrated mechanisms linking functional ingredients to gut microbiota modulation, neuroimmune signaling, and skin health outcomes are summarized in Figure 3, which illustrates how diverse compounds converge on shared axis-level pathways to produce coordinated systemic effects.

## 5. Translational and Clinical Perspectives

Although clinical evidence for many functional ingredients has been discussed within the compound-specific sections above, an integrated evaluation of their translational relevance further supports the physiological significance of gut–brain–skin axis modulation in humans. Importantly, reported clinical outcomes increasingly align with the mechanistic pathways described in Section 4 and summarized in Figure 3 and Table 2.

### 5.1. Polyphenols

Polyphenols such as quercetin, EGCG, and resveratrol have been evaluated in both dietary intervention studies and controlled clinical trials. These studies consistently report improvements in skin-related parameters, including elasticity, hydration, and wrinkle depth, alongside reductions in oxidative stress and systemic inflammatory markers. For example, supplementation with green tea catechins, rich in EGCG, has been associated with improved skin structure and protection against UV-induced damage. Similarly, resveratrol has demonstrated anti-inflammatory and metabolic regulatory effects that may indirectly support skin health through systemic pathways. Although direct measurements of gut–brain–skin axis activity remain limited, these clinical findings are consistent with mechanistic evidence linking microbiota modulation, antioxidant activation (e.g., Nrf2), and inflammatory suppression, as outlined in Figure 3 and summarized in Table 2 [53,54,55,56,73,74,75].

### 5.2. Probiotics

Among probiotics, *Lactobacillus rhamnosus* GG and related strains have demonstrated promising clinical outcomes in both dermatological and neuropsychological contexts. Randomized controlled trials have reported improvements in atopic dermatitis severity, reductions in skin inflammation, and enhancement of barrier function. In parallel, certain probiotic interventions have shown anxiolytic and stress-reducing effects, supporting modulation of the gut–brain axis. These clinical observations are consistent with mechanisms involving improved intestinal barrier integrity, reduced endotoxemia, and regulation of neuroimmune signaling pathways described in Section 4 and visualized in Figure 3 [7,99,100,101,102,103,104,105,106,107,108,109,110,111,112,113,114,115].

### 5.3. Omega-3 Fatty Acids

Omega-3 fatty acids (DHA and EPA) are among the most extensively studied compounds in clinical settings. Clinical studies have reported potential benefits in inflammatory skin disorders, including reductions in erythema, scaling, or lesion severity, although outcomes may vary by disease type, dosage, and intervention design. In addition to dermatological outcomes, omega-3 supplementation has been associated with improvements in mood and cognitive function, supporting its role in modulating gut–brain–skin interactions. These effects are mechanistically linked to the production of specialized pro-resolving mediators, suppression of pro-inflammatory cytokines, and regulation of oxidative stress pathways, as summarized in Table 2 [119,120,121,122,123,124,125,126,127,128,129,130,131,132].

### 5.4. Other Compounds (Emerging Evidence)

Additional functional compounds, including other polyphenols, peptides, and amino acid–derived metabolites, have shown preliminary clinical potential. Some studies report improvements in skin appearance and reductions in inflammatory markers; however, evidence remains limited and less consistent compared to the core compounds discussed above. Although preliminary findings are promising, many of these compounds still lack robust human clinical evidence directly evaluating gut–brain–skin axis-related outcomes. Therefore, well-designed clinical studies incorporating integrated gut, neuroimmune, and skin endpoints are required to validate their translational relevance.

### 5.5. Limitations and Future Clinical Directions

Despite encouraging findings, several limitations remain. Many clinical studies focus on single-organ outcomes rather than integrated axis-level responses, which constrains interpretation within the broader gut–brain–skin framework illustrated in Figure 1. Future trials should adopt multi-dimensional study designs incorporating microbiome profiling, neuroendocrine markers, metabolomic analysis, immune markers, and dermatological assessments to better capture system-level interactions. In addition, harmonization of study parameters, including dosage, intervention duration, formulation type, and population characteristics, will be critical for improving comparability across studies and translating functional ingredient-based interventions into consistent clinical outcomes [92,93,94,95,136,137,138,139].

From a translational perspective, the gut–brain–skin axis provides a conceptual basis for developing functional foods, nutraceuticals, cosmeceuticals, and personalized nutritional strategies for healthy skin aging. Functional foods and nutraceuticals may target upstream systemic mechanisms, including gut microbial composition, intestinal barrier integrity, microbial metabolite production, neuroendocrine balance, and low-grade inflammation. In contrast, cosmeceutical strategies primarily act at the local skin level by improving barrier function, reducing oxidative damage, supporting extracellular matrix maintenance, or modulating the cutaneous microbiome [11]. These approaches should not be viewed as mutually exclusive; rather, combined oral and topical strategies may be particularly relevant for skin aging, where systemic inflammatory tone and local cutaneous stress responses interact.

However, clinical translation requires careful distinction between direct and indirect evidence. Improvements in inflammatory skin diseases, such as atopic dermatitis or psoriasis, support the anti-inflammatory potential of probiotics, omega-3 fatty acids, and selected bioactive compounds, but they do not necessarily demonstrate anti-aging efficacy [15,16,105,131]. Aging-focused clinical trials should therefore include endpoints such as wrinkle depth, elasticity, hydration, transepidermal water loss, collagen remodeling markers, pigmentation, photoaging scores, and validated skin aging indices, together with gut microbiome, metabolomic, immune, and neuroendocrine measurements. Such integrated endpoints would allow future studies to determine whether functional ingredients truly modify the gut–brain–skin axis rather than simply improving isolated skin or inflammatory outcomes.

## 6. Challenges and Future Perspectives

Although accumulating evidence supports the relevance of the gut–brain–skin axis in human health, several important challenges must be addressed before these findings can be translated into robust clinical applications. A primary limitation lies in the heterogeneity of existing studies. Considerable variation in study design—including differences in population characteristics, intervention duration, dosage, and outcome measures—makes it difficult to directly compare results across studies or to establish standardized therapeutic strategies [5,12,92,93,94,95]. In particular, inconsistencies in endpoints related to cognitive function and skin health further complicate interpretation of clinical relevance.

Another major challenge is the complexity and inter-individual variability of the gut microbiome. Microbial composition and function are highly dynamic and are influenced by diet, age, genetic background, medication use, and environmental exposures. As a result, responses to functional ingredients can vary substantially between individuals, leading to inconsistent or context-dependent outcomes [2,3]. This variability underscores the importance of developing stratified or personalized approaches based on baseline microbiome profiles and host characteristics. In addition, a large proportion of current evidence is derived from preclinical models or small-scale human studies. While these studies provide valuable insights into underlying biological mechanisms, their direct translation to human physiology remains limited. Differences in microbial ecology, metabolism, and immune responses between experimental models and humans may lead to overestimation or underestimation of efficacy. Therefore, larger, well-designed randomized controlled trials are required to validate clinical benefits, determine optimal dosing regimens, and assess long-term safety [35,36,119,120,121,122].

From a mechanistic perspective, further work is needed to clarify causal relationships within the gut–brain–skin axis. Many studies describe associations between microbial metabolites, immune mediators, and physiological outcomes; however, the directionality and relative contribution of these pathways are not fully established. Integrative multi-omics approaches—including metagenomics, metabolomics, and transcriptomics—offer a promising strategy to address these gaps by enabling comprehensive characterization of host–microbe interactions across multiple biological layers [1]. Such approaches may facilitate the identification of key regulatory nodes and predictive biomarkers.

Another important unresolved issue is the limited integration of microbial metabolites and immune pathways. Although SCFAs are frequently discussed as major microbiota-derived mediators, additional postbiotic and microbial metabolites, including tryptophan-derived indoles, secondary bile acid derivatives, polyamines, histamine-related metabolites, microbial peptides, and lipid mediators, may also participate in gut–skin communication [140,141]. These metabolites can influence epithelial barrier function, immune cell differentiation, cytokine production, oxidative stress responses, and neuroendocrine signaling. Therefore, future studies should move beyond single-metabolite models and evaluate metabolite networks that connect microbial ecology, systemic immunity, and skin phenotypes.

Neurobehavioral factors also require greater attention. Psychological stress, anxiety, depression, and related psychiatric conditions can activate central and peripheral stress-response pathways, including the HPA axis, sympathetic nervous system, neuropeptide release, and inflammatory signaling [142,143]. These pathways may impair skin barrier function, delay repair responses, aggravate inflammatory skin diseases, and potentially accelerate skin aging through chronic inflammatory and oxidative mechanisms. Thus, brain-origin signals should not be considered secondary modifiers only; they may represent active drivers of systemic inflammation and altered skin homeostasis within the gut–brain–skin axis.

Precision-oriented approaches will also require clinically meaningful biomarkers. Candidate biomarkers may include microbiome-derived signatures, SCFA and indole metabolite profiles, bile acid patterns, circulating inflammatory cytokines, cortisol or HPA axis-related markers, oxidative stress markers, lipid mediators, extracellular vesicle-associated microRNAs, and skin phenotypes such as transepidermal water loss, hydration, elasticity, pigmentation, wrinkle depth, and dermoscopic or imaging-based features [144,145]. Multimodal stratification panels integrating clinical examination, skin imaging, microbiome profiling, metabolomics, tissue biomarkers, molecular biomarkers, and immune markers may help identify individuals who are more likely to respond to specific nutritional or functional ingredient-based interventions.

Biological sex and endocrine aging should also be considered in future studies. Menopause and andropause are accompanied by changes in sex hormone signaling that may influence skin structure, immune regulation, microbial ecology, and neuroendocrine responses. Estrogen decline, in particular, is associated with reduced collagen production, decreased elasticity, dryness, and altered barrier function, whereas age-related androgen changes may also affect sebum production, inflammation, and skin physiology [146]. These endocrine shifts may modify the response to dietary bioactives, probiotics, and omega-3 fatty acids, suggesting that sex- and age-stratified analyses are necessary for personalized intervention strategies.

Beyond these biological considerations, the gut–brain–skin axis should be interpreted within a broader lifestyle and systems-aging framework. Whole dietary patterns, food matrices, circadian rhythm, sleep quality, physical activity, stress exposure, and metabolic health may shape the axis more strongly than single compounds alone [147,148]. Emerging mechanisms, including exosome-encapsulated microRNAs, mitochondrial retrograde signaling, epigenetic regulation, and telomere homeostasis, may provide additional links between diet, microbiota, cellular metabolism, and inter-organ communication [144,145,149]. These mechanisms remain insufficiently explored in skin aging, but they offer promising directions for future multi-omics studies aimed at defining causal pathways and personalized preventive strategies.

The convergence of multiple functional ingredients on shared axis-level mechanisms also suggests that combination or multi-target approaches may provide greater efficacy than single-compound interventions. Polyphenols, probiotics, and omega-3 fatty acids may produce additive or complementary effects on microbial composition, immune regulation, oxidative stress, and neuroendocrine signaling. However, systematic evaluation of such combinatorial strategies remains limited, and their clinical relevance requires further validation.

Finally, translation into practical applications requires careful consideration of formulation, bioavailability, and regulatory factors. Many bioactive compounds exhibit limited stability or absorption, which may reduce their effectiveness in vivo. Advances in delivery systems, including encapsulation technologies and targeted release platforms, may help overcome these limitations and improve therapeutic potential. In parallel, regulatory frameworks must evolve to support the development of evidence-based functional products targeting complex biological systems. Addressing these challenges will be essential for advancing the gut–brain–skin axis from a conceptual framework toward a clinically actionable target for promoting healthy aging, cognitive resilience, and skin health.

## 7. Conclusions

This review highlights the emerging role of the gut–brain–skin axis as an integrative framework linking gastrointestinal function, neuroimmune signaling, and skin physiology. Across the studies examined, diverse functional ingredients—including polyphenols, probiotics, and omega-3 fatty acids—demonstrate the capacity to influence this axis through interconnected biological pathways. Despite their structural diversity, these compounds consistently converge on key mechanisms, such as modulation of gut microbiota composition, reinforcement of intestinal barrier integrity, regulation of immune responses, and attenuation of oxidative stress. Importantly, the beneficial effects of orally administered functional ingredients on skin health may not be fully explained by direct accumulation within skin tissues alone, but rather by coordinated systemic regulation across the gut–brain–skin axis.

Furthermore, these gut-derived effects extend beyond the local intestinal environment. Microbial metabolites and immune mediators can influence central nervous system function through neural, endocrine, and circulatory pathways, which in turn shape skin physiology by modulating inflammatory responses, barrier function, and tissue remodeling processes [1,2,3]. This bidirectional and system-level interaction underscores the relevance of the gut–brain–skin axis in the context of aging and chronic inflammatory conditions.

Looking forward, the convergence of multiple functional ingredients on shared biological pathways suggests that multi-target or combination-based strategies may offer enhanced efficacy compared to single-compound interventions. However, future research should move beyond descriptive pathway summaries and more clearly distinguish direct evidence for healthy skin aging from indirect evidence derived from inflammatory skin diseases. Integrated clinical trials incorporating microbiome profiling, metabolomics, neuroendocrine and immune markers, skin imaging, and validated dermatological endpoints will be essential for determining whether modulation of the gut–brain–skin axis can produce clinically meaningful benefits.

In conclusion, modulation of the gut–brain–skin axis represents a promising and biologically plausible approach for promoting healthy skin aging, maintaining cognitive resilience, and improving systemic inflammatory balance. The next stage of research should integrate functional foods, nutraceuticals, cosmeceuticals, lifestyle interventions, and precision nutrition within a multimodal framework. Such approaches may help translate the gut–brain–skin axis from a conceptual model into predictive, preventive, and personalized strategies for healthy aging.

## Figures and Tables

**Table 1:** Comparison of systemic inflammation in healthy skin aging and common inflammatory skin diseases. Healthy skin aging is mainly characterized by chronic low-grade inflammation and gradual tissue remodeling, whereas common inflammatory skin diseases such as atopic dermatitis and psoriasis involve more disease-specific immune pathways. This comparison distinguishes aging-related inflammatory mechanisms from disease-associated inflammatory responses and clarifies how evidence from inflammatory skin diseases may provide indirect, but not equivalent, translational support for healthy skin aging [14,15,16].

Condition	Inflammatory Pattern and Key Mediators	Clinical Features and Outcomes	Interpretation for the Gut–Brain–Skin Axis
Healthy skin aging	Chronic low-grade inflammation, often described as inflammaging, involving IL-6, TNF-α, IL-1β, ROS, MMP activation, mitochondrial dysfunction, and senescence-associated signaling	Wrinkles, reduced elasticity, dryness, collagen degradation, impaired barrier function, and increased transepidermal water loss	Gut dysbiosis, reduced SCFA production, increased intestinal permeability, HPA axis dysregulation, and neuroimmune signaling may contribute to systemic aging-related skin decline. Direct aging evidence remains emerging, and many mechanisms are supported by preclinical or indirect clinical data.
Atopic dermatitis/eczema	Type 2-skewed inflammation and barrier dysfunction involving IL-4, IL-13, IL-31, TSLP, altered skin microbiome, and impaired epithelial barrier signaling	Pruritus, eczematous lesions, recurrent inflammation, and barrier disruption	Gut dysbiosis, altered immune tolerance, stress, and neuroimmune signaling may aggravate disease severity. Evidence from atopic dermatitis provides useful inflammatory-disease support but should not be interpreted as direct evidence for healthy skin aging.
Psoriasis	IL-23/Th17-driven systemic inflammation involving IL-17, IL-23, TNF-α, keratinocyte hyperproliferation, and systemic inflammatory mediators	Erythematous plaques, scaling, epidermal thickening, and systemic comorbidity risk	Gut and skin microbiome alterations may contribute to systemic immune activation and inflammatory amplification. These mechanisms are informative for systemic inflammation but differ from gradual skin aging.
Primarily cutaneous /photoaging	Local tissue damage and cutaneous inflammatory signaling involving UV-induced ROS, AP-1, NF-κB, MMPs, collagen breakdown, and fibroblast senescence	Photoaging, pigmentation, wrinkles, and dermal matrix degradation	This process is mainly skin-driven, although systemic inflammation may modify tissue resilience and repair capacity. It is primarily addressed by photoprotection and dermatological interventions, with possible complementary systemic support.

**Table 2:** Representative food-derived functional ingredients targeting the gut–brain–skin axis and their primary mechanistic and translational evidence. Compounds in Table 2A were selected for in-depth analysis because they are discussed in detail in Section 4 and show relatively integrated evidence across gut microbiota modulation, neuroimmune or neuroendocrine signaling, and skin-related outcomes. Compounds in Table 2B are additional functional ingredients discussed in Section 4 that may contribute to the gut–brain–skin axis, but their current evidence is more limited, indirect, or focused on specific domains such as skin outcomes, gut–brain signaling, inflammation, or strain-specific probiotic effects. Evidence descriptors therefore indicate the primary focus and maturity of available evidence rather than direct comparative clinical strength. SCFAs are not listed as independent functional ingredients because they are microbiota-derived metabolites and mechanistic mediators within the axis rather than dietary intervention compounds.

**A. Deep Dive Compounds**				
**Category**	**Compound**	**Key Mechanism (Gut → Brain → Skin)**	**Evidence Level**	**Ref**
Polyphenols (Flavonols)	Quercetin	Enhances beneficial microbiota and SCFA production → suppresses NF-κB–mediated neuroinflammation and oxidative stress → inhibits MMP activity and improves skin barrier integrity	Preclinical, Emerging Clinical	[5,12,13,18,19,20,21,22,23,24,25,26,27,28,29,30,31,32,33,34,35,36,37,38]
Polyphenols (Flavanols)	EGCG	Modulates gut microbiota and strengthens intestinal barrier → inhibits NF-κB signaling and microglial activation → reduces UV-induced inflammation and collagen degradation	Preclinical, Emerging Clinical	[39,40,41,42,43,44,45,46,47,48,49,50,51,52,53,54,55,56]
Polyphenols (Stilbenes)	Resveratrol	Reshapes microbiota and reduces endotoxemia → activates SIRT1/AMPK and regulates HPA axis → promotes collagen synthesis and attenuates oxidative skin damage	Preclinical, Emerging Clinical	[10,24,25,26,57,58,59,60,61,62,63,64,65,66,67,68,69,70,71,72,73,74,75]
Probiotics (*Lactobacillus*)	*Lactobacillus rhamnosus* GG	Enhances gut barrier and reduces LPS translocation → modulates gut–brain stress-related and neuroimmune signaling → may improve skin inflammation and atopic dermatitis-related outcomes	RCT-supported, strain-specific evidence	[7,96,97,98,99,100,101,102,103,104,105,106,107,108,109,110,111,112,113,114,115]
Omega-3 fatty acids	DHA/EPA	Improves gut microbial balance and reduces inflammation → generates pro-resolving mediators and attenuates neuroinflammation → enhances skin barrier repair and reduces inflammatory skin responses	Clinical evidence, skin-inflammation-focused	[119,120,121,122,123,124,125,126,127,128,129,130,131,132]
**B. Additional Compounds**				
**Category**	**Compound**	**Main Function**	**Evidence Level**	**Ref**
Polyphenols (Flavonols)	Kaempferol	Antioxidant and anti-inflammatory effects; modulation of gut microbiota	Mechanistic, preclinical	[80,81]
Polyphenols (Anthocyanins)	Cyanidin/Anthocyanins	UV protection, antioxidant activity, enhancement of microbial diversity	Skin- and microbiota-focused supportive evidence	[82,83,84,85]
Polyphenols (Curcuminoids)	Curcumin	NF-κB inhibition, anti-inflammatory effects, gut microbiota modulation	Inflammation- and gut-modulation-focused evidence	[86,87,88,89,90,91]
Probiotics	*Bifidobacterium longum*/other probiotic strains	Immune modulation, stress-response regulation, and skin-related benefits with strain-specific variability	Strain-specific, variable evidence	[92,93,94,95,116,117,118]
Peptides	Collagen peptides	Support dermal extracellular matrix synthesis and improve skin elasticity	Skin-focused RCT evidence	[133,134,135]
Amino acid-derived metabolites	Tryptophan-derived metabolites/indole derivatives	Modulate gut–brain signaling, neurotransmitter pathways, and immune responses	Gut–brain-focused preclinical evidence	[17,136,137,138,139]

Abbreviations: AMPK, AMP-activated protein kinase; DHA, docosahexaenoic acid; EGCG, epigallocatechin-3-gallate; EPA, eicosapentaenoic acid; HPA, hypothalamic–pituitary–adrenal; LPS, lipopolysaccharide; MMP, matrix metalloproteinase; NF-κB, nuclear factor kappa B; RCT, randomized controlled trial; SCFA(s), short-chain fatty acid(s); SIRT1, sirtuin 1; UV, ultraviolet.

## Data Availability

No new data were created or analyzed in this study. Data sharing is not applicable to this article.

## References

[1] Liu P., Ouyang Y., Gao Z., Tan J., Chen X., Xiao Y., Wang Y., Liu J., Liu B., Gao B. (2025). Decoding the Gut-Brain Axis in Depression: Mechanistic Insights and Functional Microbiota-Based Interventions. J. Funct. Foods.

[2] O’Neill C.A., Monteleone G., McLaughlin J.T., Paus R. (2016). The Gut-Skin Axis in Health and Disease: A Paradigm with Therapeutic Implications. BioEssays.

[3] Salem I., Ramser A., Isham N., Ghannoum M.A. (2018). The Gut Microbiome as a Major Regulator of the Gut-Skin Axis. Front. Microbiol..

[4] Li Y., Li T., Zhang Y., Wang Y., Wu G., Tan Y. (2026). Microbial Metabolites in the Gut-Brain Axis: Their Impact on Depression Pathophysiology and Treatment. Neuroscience.

[5] Koh A., Vadder F.D., Kovatcheva-Datchary P., Bäckhed F. (2016). From Dietary Fiber to Host Physiology: Short-Chain Fatty Acids as Key Bacterial Metabolites. Cell.

[6] Lee A., Lee J.Y., Jung S.W., Shin S.Y., Ryu H.S., Jang S.-H., Kwon J.G., Kim Y.S. (2023). Brain–Gut–Microbiota Axis. Korean J. Gastroenterol..

[7] Marano G., Mazza M., Lisci F.M., Ciliberto M., Traversi G., Kotzalidis G.D., De Berardis D., Laterza L., Sani G., Gasbarrini A. (2023). The Microbiota-Gut-Brain Axis: Psychoneuroimmunological Insights. Nutrients.

[8] Sharon G., Sampson T.R., Geschwind D.H., Mazmanian S.K. (2016). The Central Nervous System and the Gut Microbiome. Cell.

[9] Ataei P., Kalantari H., Bodnar T.S., Turner R.J. (2026). The Gut–Brain Connection: Microbes’ Influence on Mental Health and Psychological Disorders. Front. Microbiomes.

[10] Chung J.Y., Jeong J.-H., Song J. (2020). Resveratrol Modulates the Gut-Brain Axis: Focus on Glucagon-Like Peptide-1, 5-HT, and Gut Microbiota. Front. Aging Neurosci..

[11] Woo Y.R., Kim H.S. (2024). Interaction between the Microbiota and the Skin Barrier in Aging Skin: A Comprehensive Review. Front. Physiol..

[12] Marek-Jozefowicz L., Nedoszytko B., Grochocka M., Żmijewski M.A., Czajkowski R., Cubała W.J., Slominski A.T. (2023). Molecular Mechanisms of Neurogenic Inflammation of the Skin. Int. J. Mol. Sci..

[13] Li Y., Yao J., Han C., Yang J., Chaudhry M.T., Wang S., Liu H., Yin Y. (2016). Quercetin, Inflammation and Immunity. Nutrients.

[14] Franceschi C., Garagnani P., Parini P., Giuliani C., Santoro A. (2018). Inflammaging: A New Immune–Metabolic Viewpoint for Age-Related Diseases. Nat. Rev. Endocrinol..

[15] Kim J., Kim B.E., Leung D.Y.M. (2019). Pathophysiology of Atopic Dermatitis: Clinical Implications. Allergy Asthma Proc..

[16] Liu T., Li S., Ying S., Tang S., Ding Y., Li Y., Qiao J., Fang H. (2020). The IL-23/IL-17 Pathway in Inflammatory Skin Diseases: From Bench to Bedside. Front. Immunol..

[17] Agus A., Planchais J., Sokol H. (2018). Gut Microbiota Regulation of Tryptophan Metabolism in Health and Disease. Cell Host Microbe.

[18] Xu D., Hu M.-J., Wang Y.-Q., Cui Y.-L. (2019). Antioxidant Activities of Quercetin and Its Complexes for Medicinal Application. Molecules.

[19] Zhang H.-X., Li Y.-Y., Liu Z.-J., Wang J.-F. (2022). Quercetin Effectively Improves LPS-Induced Intestinal Inflammation, Pyroptosis, and Disruption of the Barrier Function through the TLR4/NF-κB/NLRP3 Signaling Pathway in Vivo and in Vitro. Food Nutr. Res..

[20] Li H., Gao J., Peng W., Sun X., Qi W., Wang Y. (2025). Dietary Polyphenols-Gut Microbiota Interactions: Intervention Strategies and Metabolic Regulation for Intestinal Diseases. Biology.

[21] Wang X., Qi Y., Zheng H. (2022). Dietary Polyphenol, Gut Microbiota, and Health Benefits. Antioxidants.

[22] Plamada D., Vodnar D.C. (2021). Polyphenols—Gut Microbiota Interrelationship: A Transition to a New Generation of Prebiotics. Nutrients.

[23] Ross F.C., Patangia D., Grimaud G., Lavelle A., Dempsey E.M., Ross R.P., Stanton C. (2024). The Interplay between Diet and the Gut Microbiome: Implications for Health and Disease. Nat. Rev. Microbiol..

[24] Güemes-González A.M., Arriaga-Pizano L.A., Chacón-Salinas R., Wong-Baeza I., Ferat-Osorio E. (2026). Regulation of Intestinal Permeability in Health and Disease: Possible Therapeutic Applications. Arch. Med. Res..

[25] Ferris M.M., Subitoni Antonio L., Al-Sadi R. (2025). Probiotics and the Intestinal Tight Junction Barrier Function. Front. Cell Dev. Biol..

[26] Camilleri M. (2019). Leaky Gut: Mechanisms, Measurement and Clinical Implications in Humans. Gut.

[27] Aggarwal D., Chaudhary M., Mandotra S.K., Tuli H.S., Chauhan R., Joshi N.C., Kaur D., Dufossé L., Chauhan A. (2025). Anti-Inflammatory Potential of Quercetin: From Chemistry and Mechanistic Insight to Nanoformulations. Curr. Res. Pharmacol. Drug Discov..

[28] Mamun A.A., Shao C., Geng P., Wang S., Xiao J. (2024). Polyphenols Targeting NF-κB Pathway in Neurological Disorders: What We Know So Far?. Int. J. Biol. Sci..

[29] Liu Y., Jiao A. (2025). Flavonoids as Immunoregulators: Molecular Mechanisms in Regulating Immune Cells and Their Therapeutic Applications in Inflammatory Diseases. Front. Immunol..

[30] Ngo V., Duennwald M.L. (2022). Nrf2 and Oxidative Stress: A General Overview of Mechanisms and Implications in Human Disease. Antioxidants.

[31] Lin L., Wu Q., Lu F., Lei J., Zhou Y., Liu Y., Zhu N., Yu Y., Ning Z., She T. (2023). Nrf2 Signaling Pathway: Current Status and Potential Therapeutic Targetable Role in Human Cancers. Front. Oncol..

[32] Lee J.-S., Min J.-W., Gye S.-B., Kim Y.-W., Kang H.-C., Choi Y.-S., Seo W.-S., Lee B.-Y. (2024). Suppression of UVB-Induced MMP-1 Expression in Human Skin Fibroblasts Using Lysate of Lactobacillus Iners Derived from Korean Women’s Skin in Their Twenties. Curr. Issues Mol. Biol..

[33] Feng C., Chen X., Yin X., Jiang Y., Zhao C. (2024). Matrix Metalloproteinases on Skin Photoaging. J. Cosmet. Dermatol..

[34] Lai C.-F., Esam G., Lin Y.-C., Chung R.-J. (2026). From Destruction to Protection: Rethinking MMP-1 in Skin Aging and Anti-Aging Strategies. Arch. Dermatol. Res..

[35] Li Y., Tian X., Luo J., Bao T., Wang S., Wu X. (2024). Molecular Mechanisms of Aging and Anti-Aging Strategies. Cell Commun. Signal..

[36] Addor F.A.S. (2017). Antioxidants in Dermatology. An. Bras. Dermatol..

[37] Mirza M.A., Mahmood S., Hilles A.R., Ali A., Khan M.Z., Zaidi S.A.A., Iqbal Z., Ge Y. (2023). Quercetin as a Therapeutic Product: Evaluation of Its Pharmacological Action and Clinical Applications-A Review. Pharmaceuticals.

[38] Aghababaei F., Hadidi M. (2023). Recent Advances in Potential Health Benefits of Quercetin. Pharmaceuticals.

[39] Yan Z., Zhong Y., Duan Y., Chen Q., Li F. (2020). Antioxidant Mechanism of Tea Polyphenols and Its Impact on Health Benefits. Anim. Nutr..

[40] Mokra D., Joskova M., Mokry J. (2022). Therapeutic Effects of Green Tea Polyphenol (‒)-Epigallocatechin-3-Gallate (EGCG) in Relation to Molecular Pathways Controlling Inflammation, Oxidative Stress, and Apoptosis. Int. J. Mol. Sci..

[41] Clarke K.A., Dew T.P., Watson R.E.B., Farrar M.D., Osman J.E., Nicolaou A., Rhodes L.E., Williamson G. (2016). Green Tea Catechins and Their Metabolites in Human Skin before and after Exposure to Ultraviolet Radiation. J. Nutr. Biochem..

[42] Yang J., Chen W., Chen J., Xie D., Wang Y., Zhou J. (2025). Tea Polyphenol Epigallocatechin Gallate and the Gut-Health Axis: Unraveling Structural Characteristics, Metabolic Pathways, and Systemic Benefits. Adv. Nutr..

[43] Wu Z., Shen J., Xu Q., Xiang Q., Chen Y., Lv L., Zheng B., Wang Q., Wang S., Li L. (2022). Epigallocatechin-3-Gallate Improves Intestinal Gut Microbiota Homeostasis and Ameliorates Clostridioides Difficile Infection. Nutrients.

[44] Payne A., Taka E., Adinew G.M., Soliman K.F.A. (2023). Molecular Mechanisms of the Anti-Inflammatory Effects of Epigallocatechin 3-Gallate (EGCG) in LPS-Activated BV-2 Microglia Cells. Brain Sci..

[45] Liu C., Liu J., Wang W., Yang M., Chi K., Xu Y., Guo N. (2023). Epigallocatechin Gallate Alleviates Staphylococcal Enterotoxin A-Induced Intestinal Barrier Damage by Regulating Gut Microbiota and Inhibiting the TLR4-NF-κB/MAPKs-NLRP3 Inflammatory Cascade. J. Agric. Food Chem..

[46] Bischoff S.C., Barbara G., Buurman W., Ockhuizen T., Schulzke J.-D., Serino M., Tilg H., Watson A., Wells J.M. (2014). Intestinal Permeability—A New Target for Disease Prevention and Therapy. BMC Gastroenterol..

[47] Payne A., Nahashon S., Taka E., Adinew G.M., Soliman K.F.A. (2022). Epigallocatechin-3-Gallate (EGCG): New Therapeutic Perspectives for Neuroprotection, Aging, and Neuroinflammation for the Modern Age. Biomolecules.

[48] Zhong X., Liu M., Yao W., Du K., He M., Jin X., Jiao L., Ma G., Wei B., Wei M. (2019). Epigallocatechin-3-Gallate Attenuates Microglial Inflammation and Neurotoxicity by Suppressing the Activation of Canonical and Noncanonical Inflammasome via TLR4/NF-κB Pathway. Mol. Nutr. Food Res..

[49] Farooqi A.A., Pinheiro M., Granja A., Farabegoli F., Reis S., Attar R., Sabitaliyevich U.Y., Xu B., Ahmad A. (2020). EGCG Mediated Targeting of Deregulated Signaling Pathways and Non-Coding RNAs in Different Cancers: Focus on JAK/STAT, Wnt/β-Catenin, TGF/SMAD, NOTCH, SHH/GLI, and TRAIL Mediated Signaling Pathways. Cancers.

[50] Kim E., Hwang K., Lee J., Han S.Y., Kim E.-M., Park J., Cho J.Y. (2018). Skin Protective Effect of Epigallocatechin Gallate. Int. J. Mol. Sci..

[51] Sun J., Jiang Y., Fu J., He L., Guo X., Ye H., Yin C., Li H., Jiang H. (2024). Beneficial Effects of Epigallocatechin Gallate in Preventing Skin Photoaging: A Review. Molecules.

[52] Mereles D., Hunstein W. (2011). Epigallocatechin-3-Gallate (EGCG) for Clinical Trials: More Pitfalls than Promises?. Int. J. Mol. Sci..

[53] Di Sotto A., Gullì M., Percaccio E., Vitalone A., Mazzanti G., Di Giacomo S. (2022). Efficacy and Safety of Oral Green Tea Preparations in Skin Ailments: A Systematic Review of Clinical Studies. Nutrients.

[54] Domingo D.S., Camouse M.M., Hsia A.H., Matsui M., Maes D., Ward N.L., Cooper K.D., Baron E.D. (2010). Anti-Angiogenic Effects of Epigallocatechin-3-Gallate in Human Skin. Int. J. Clin. Exp. Pathol..

[55] Nagle D.G., Ferreira D., Zhou Y.-D. (2006). Epigallocatechin-3-Gallate (EGCG): Chemical and Biomedical Perspectives. Phytochemistry.

[56] Ferrari E., Naponelli V. (2025). Catechins and Human Health: Breakthroughs from Clinical Trials. Molecules.

[57] Singh A.P., Singh R., Verma S.S., Rai V., Kaschula C.H., Maiti P., Gupta S.C. (2019). Health Benefits of Resveratrol: Evidence from Clinical Studies. Med. Res. Rev..

[58] Zhang L.-X., Li C.-X., Kakar M.U., Khan M.S., Wu P.-F., Amir R.M., Dai D.-F., Naveed M., Li Q.-Y., Saeed M. (2021). Resveratrol (RV): A Pharmacological Review and Call for Further Research. Biomed. Pharmacother..

[59] Yu X., Jia Y., Ren F. (2024). Multidimensional Biological Activities of Resveratrol and Its Prospects and Challenges in the Health Field. Front. Nutr..

[60] Ren Z., Zheng S., Sun Z., Luo Y., Wang Y., Yi P., Li Y., Huang C., Xiao W. (2025). Resveratrol: Molecular Mechanisms, Health Benefits, and Potential Adverse Effects. MedComm.

[61] Chaplin A., Carpéné C., Mercader J. (2018). Resveratrol, Metabolic Syndrome, and Gut Microbiota. Nutrients.

[62] Wang Y., Hong C., Wu Z., Li S., Xia Y., Liang Y., He X., Xiao X., Tang W. (2022). Resveratrol in Intestinal Health and Disease: Focusing on Intestinal Barrier. Front. Nutr..

[63] Cai T.-T., Ye X.-L., Li R.-R., Chen H., Wang Y.-Y., Yong H.-J., Pan M.-L., Lu W., Tang Y., Miao H. (2020). Resveratrol Modulates the Gut Microbiota and Inflammation to Protect Against Diabetic Nephropathy in Mice. Front. Pharmacol..

[64] Rogina B., Tissenbaum H.A. (2024). SIRT1, Resveratrol and Aging. Front. Genet..

[65] Yang X., Jiang T., Wang Y., Guo L. (2019). The Role and Mechanism of SIRT1 in Resveratrol-Regulated Osteoblast Autophagy in Osteoporosis Rats. Sci. Rep..

[66] Lan F., Weikel K.A., Cacicedo J.M., Ido Y. (2017). Resveratrol-Induced AMP-Activated Protein Kinase Activation Is Cell-Type Dependent: Lessons from Basic Research for Clinical Application. Nutrients.

[67] Yang X.-H., Song S.-Q., Xu Y. (2017). Resveratrol Ameliorates Chronic Unpredictable Mild Stress-Induced Depression-like Behavior: Involvement of the HPA Axis, Inflammatory Markers, BDNF, and Wnt/β-Catenin Pathway in Rats. Neuropsychiatr. Dis. Treat..

[68] Rahman M.H., Akter R., Bhattacharya T., Abdel-Daim M.M., Alkahtani S., Arafah M.W., Al-Johani N.S., Alhoshani N.M., Alkeraishan N., Alhenaky A. (2020). Resveratrol and Neuroprotection: Impact and Its Therapeutic Potential in Alzheimer’s Disease. Front. Pharmacol..

[69] Cicero A.F.G., Ruscica M., Banach M. (2019). Resveratrol and Cognitive Decline: A Clinician Perspective. Arch. Med. Sci..

[70] Hecker A., Schellnegger M., Hofmann E., Luze H., Nischwitz S.P., Kamolz L., Kotzbeck P. (2021). The Impact of Resveratrol on Skin Wound Healing, Scarring, and Aging. Int. Wound J..

[71] Lin M.-H., Hung C.-F., Sung H.-C., Yang S.-C., Yu H.-P., Fang J.-Y. (2021). The Bioactivities of Resveratrol and Its Naturally Occurring Derivatives on Skin. J. Food Drug Anal..

[72] Zhou D.-D., Luo M., Huang S.-Y., Saimaiti A., Shang A., Gan R.-Y., Li H.-B. (2021). Effects and Mechanisms of Resveratrol on Aging and Age-Related Diseases. Oxid. Med. Cell Longev..

[73] Walle T. (2011). Bioavailability of Resveratrol. Ann. N. Y. Acad. Sci..

[74] Ramírez-Garza S.L., Laveriano-Santos E.P., Marhuenda-Muñoz M., Storniolo C.E., Tresserra-Rimbau A., Vallverdú-Queralt A., Lamuela-Raventós R.M. (2018). Health Effects of Resveratrol: Results from Human Intervention Trials. Nutrients.

[75] Movahed A., Raj P., Nabipour I., Mahmoodi M., Ostovar A., Kalantarhormozi M., Netticadan T. (2020). Efficacy and Safety of Resveratrol in Type 1 Diabetes Patients: A Two-Month Preliminary Exploratory Trial. Nutrients.

[76] Khoo H.E., Azlan A., Tang S.T., Lim S.M. (2017). Anthocyanidins and Anthocyanins: Colored Pigments as Food, Pharmaceutical Ingredients, and the Potential Health Benefits. Food Nutr. Res..

[77] Jokioja J., Yang B., Linderborg K.M. (2021). Acylated Anthocyanins: A Review on Their Bioavailability and Effects on Postprandial Carbohydrate Metabolism and Inflammation. Compr. Rev. Food Sci. Food Saf..

[78] Cory H., Passarelli S., Szeto J., Tamez M., Mattei J. (2018). The Role of Polyphenols in Human Health and Food Systems: A Mini-Review. Front. Nutr..

[79] Vauzour D., Rodriguez-Mateos A., Corona G., Oruna-Concha M.J., Spencer J.P.E. (2010). Polyphenols and Human Health: Prevention of Disease and Mechanisms of Action. Nutrients.

[80] Suzuki T., Tanabe S., Hara H. (2011). Kaempferol Enhances Intestinal Barrier Function through the Cytoskeletal Association and Expression of Tight Junction Proteins in Caco-2 Cells. J. Nutr..

[81] Bian Y., Lei J., Zhong J., Wang B., Wan Y., Li J., Liao C., He Y., Liu Z., Ito K. (2022). Kaempferol Reduces Obesity, Prevents Intestinal Inflammation, and Modulates Gut Microbiota in High-Fat Diet Mice. J. Nutr. Biochem..

[82] Diaconeasa Z., Știrbu I., Xiao J., Leopold N., Ayvaz Z., Danciu C., Ayvaz H., Stǎnilǎ A., Nistor M., Socaciu C. (2020). Anthocyanins, Vibrant Color Pigments, and Their Role in Skin Cancer Prevention. Biomedicines.

[83] Yu J., Zhang Z., Jiang H., Pu H., Xiong Y., Fan Y., Li K., Yu X., Zhu Y. (2026). Anthocyanins in Cancer Prevention: A Bibliometric Review. Nat. Prod. Commun..

[84] Bae J.-Y., Lim S.S., Kim S.J., Choi J.-S., Park J., Ju S.M., Han S.J., Kang I.-J., Kang Y.-H. (2009). Bog Blueberry Anthocyanins Alleviate Photoaging in Ultraviolet-B Irradiation-Induced Human Dermal Fibroblasts. Mol. Nutr. Food Res..

[85] Liang A., Leonard W., Beasley J.T., Fang Z., Zhang P., Ranadheera C.S. (2024). Anthocyanins-Gut Microbiota-Health Axis: A Review. Crit. Rev. Food Sci. Nutr..

[86] Hewlings S.J., Kalman D.S. (2017). Curcumin: A Review of Its’ Effects on Human Health. Foods.

[87] Yadav R., Mishra S., Chaturvedi R., Pandey A. (2025). Therapeutic Potential of Curcumin in Cardiovascular Disease: Targeting Atherosclerosis Pathophysiology. Biomed. Pharmacother..

[88] Islam M.R., Rauf A., Akash S., Trisha S.I., Nasim A.H., Akter M., Dhar P.S., Ogaly H.A., Hemeg H.A., Wilairatana P. (2024). Targeted Therapies of Curcumin Focus on Its Therapeutic Benefits in Cancers and Human Health: Molecular Signaling Pathway-Based Approaches and Future Perspectives. Biomed. Pharmacother..

[89] Hsu K.-Y., Majeed A., Ho C.-T., Pan M.-H. (2025). Bisdemethoxycurcumin and Curcumin Alleviate Inflammatory Bowel Disease by Maintaining Intestinal Epithelial Integrity and Regulating Gut Microbiota in Mice. J. Agric. Food Chem..

[90] Wang J., Ghosh S.S., Ghosh S. (2017). Curcumin Improves Intestinal Barrier Function: Modulation of Intracellular Signaling, and Organization of Tight Junctions. Am. J. Physiol. Cell Physiol..

[91] Scazzocchio B., Minghetti L., D’Archivio M. (2020). Interaction between Gut Microbiota and Curcumin: A New Key of Understanding for the Health Effects of Curcumin. Nutrients.

[92] Marco M.L., Cunningham M., Bischoff S.C., Clarke G., Delzenne N., Lewis J.D., Meisel M., Merenstein D., O’Toole P.W., Staudacher H.M. (2026). The International Scientific Association for Probiotics and Prebiotics (ISAPP) Consensus Statement on the Definition and Scope of Gut Health. Nat. Rev. Gastroenterol. Hepatol..

[93] Smolinska S., Popescu F.-D., Zemelka-Wiacek M. (2025). A Review of the Influence of Prebiotics, Probiotics, Synbiotics, and Postbiotics on the Human Gut Microbiome and Intestinal Integrity. J. Clin. Med..

[94] Plaza-Diaz J., Ruiz-Ojeda F.J., Gil-Campos M., Gil A. (2019). Mechanisms of Action of Probiotics. Adv. Nutr..

[95] Sarita B., Samadhan D., Hassan M.Z., Kovaleva E.G. (2025). A Comprehensive Review of Probiotics and Human Health-Current Prospective and Applications. Front. Microbiol..

[96] Leser T., Baker A. (2024). Molecular Mechanisms of Lacticaseibacillus Rhamnosus, LGG^®^ Probiotic Function. Microorganisms.

[97] Aponte M., Murru N., Shoukat M. (2020). Therapeutic, Prophylactic, and Functional Use of Probiotics: A Current Perspective. Front. Microbiol..

[98] Mahalak K.K., Firrman J., Bobokalonov J., Narrowe A.B., Bittinger K., Daniel S., Tanes C., Mattei L.M., Zeng W.-B., Soares J.W. (2022). Persistence of the Probiotic Lacticaseibacillus Rhamnosus Strain GG (LGG) in an In Vitro Model of the Gut Microbiome. Int. J. Mol. Sci..

[99] Gui L., Duan X., Wang H., Xie H., Zhang R., Jiang W., Tang W. (2025). Lactobacillus Rhamnosus GG Maintains Gut Microbiota Stability and Promotes Intestinal Adaptation via Activated Intestinal Farnesoid X Receptor Signaling in Short Bowel Syndrome. Commun. Biol..

[100] Nermes M., Kantele J.M., Atosuo T.J., Salminen S., Isolauri E. (2011). Interaction of Orally Administered *Lactobacillus rhamnosus* GG with Skin and Gut Microbiota and Humoral Immunity in Infants with Atopic Dermatitis. Clin. Exp. Allergy.

[101] Chen J., Chen X., Ho C.L. (2021). Recent Development of Probiotic Bifidobacteria for Treating Human Diseases. Front. Bioeng. Biotechnol..

[102] Mehta I., Juneja K., Nimmakayala T., Bansal L., Pulekar S., Duggineni D., Ghori H.K., Modi N., Younas S. (2025). Gut Microbiota and Mental Health: A Comprehensive Review of Gut-Brain Interactions in Mood Disorders. Cureus.

[103] Wan M., Liu W., Liu Y., Jiang X., Jiang S., Duan J. (2025). The Microbiota-Gut-Brain Axis Theory: A New Perspective to Unlock the Pathological Mechanism of Constipation. Pathol. Res. Pract..

[104] Bravo J.A., Forsythe P., Chew M.V., Escaravage E., Savignac H.M., Dinan T.G., Bienenstock J., Cryan J.F. (2011). Ingestion of Lactobacillus Strain Regulates Emotional Behavior and Central GABA Receptor Expression in a Mouse via the Vagus Nerve. Proc. Natl. Acad. Sci. USA.

[105] Arif M.I., Dai Q., Ru L. (2026). Probiotics for Pediatric Atopic Dermatitis: A Systematic Review and Meta-Analysis of Randomized Controlled Trials. J. Allergy Clin. Immunol. Glob..

[106] Gao T., Wang X., Li Y., Ren F. (2023). The Role of Probiotics in Skin Health and Related Gut–Skin Axis: A Review. Nutrients.

[107] Gowda V., Sarkar R., Verma D., Das A. (2024). Probiotics in Dermatology: An Evidence-Based Approach. Indian Dermatol. Online J..

[108] Li Z., Zhang J., Zhang Y., Chen H., Bao Y. (2025). Skin Microbiome in Health and Disease: Mechanisms and Emerging Therapeutic Strategies. Clin. Cosmet. Investig. Dermatol..

[109] Nowicka D., Kucharczyk E., Pawłuszkiewicz K., Korgiel M., Busłowicz T., Ponikowska M. (2025). Topical Probiotics as a Novel Approach in the Treatment of Chronic Dermatoses Associated with Skin Dysbiosis: A Narrative Review. Int. J. Mol. Sci..

[110] Munteanu C., Turti S., Marza S.M. (2025). Unraveling the Gut–Skin Axis: The Role of Microbiota in Skin Health and Disease. Cosmetics.

[111] Ferraretto A., Donetti E., García-Mena J., Pacheco-López G. (2023). Editorial: The Gut-Skin-Brain Axis in Human Health and Disease. Front. Nutr..

[112] Zhao Y., Yu C., Zhang J., Yao Q., Zhu X., Zhou X. (2025). The Gut-skin Axis: Emerging Insights in Understanding and Treating Skin Diseases through Gut Microbiome Modulation (Review). Int. J. Mol. Med..

[113] MacGibeny M.A., Adjei S., Pyle H., Bunick C.G., Ghannoum M., Grada A., Harris-Tryon T., Tyring S.K., Kong H.H. (2025). The Human Skin Microbiome in Health. J. Am. Acad. Dermatol..

[114] Fölster-Holst R., Müller F., Schnopp N., Abeck D., Kreiselmaier I., Lenz T., von Rüden U., Schrezenmeir J., Christophers E., Weichenthal M. (2006). Prospective, Randomized Controlled Trial on *Lactobacillus rhamnosus* in Infants with Moderate to Severe Atopic Dermatitis. Br. J. Dermatol..

[115] Grüber C., Wendt M., Sulser C., Lau S., Kulig M., Wahn U., Werfel T., Niggemann B. (2007). Randomized, Placebo-Controlled Trial of *Lactobacillus rhamnosus* GG as Treatment of Atopic Dermatitis in Infancy. Allergy.

[116] Mills S., Yang B., Smith G.J., Stanton C., Ross R.P. (2023). Efficacy of *Bifidobacterium longum* Alone or in Multi-Strain Probiotic Formulations during Early Life and Beyond. Gut Microbes.

[117] Kim S., Han S.-Y., Lee J., Kim N.-R., Lee B.R., Kim H., Kwon M., Ahn K., Noh Y., Kim S.J. (2022). *Bifidobacterium longum* and Galactooligosaccharide Improve Skin Barrier Dysfunction and Atopic Dermatitis-like Skin. Allergy Asthma Immunol. Res..

[118] Boehme M., Rémond-Derbez N., Lerond C., Lavalle L., Keddani S., Steinmann M., Rytz A., Dalile B., Verbeke K., Van Oudenhove L. (2023). *Bifidobacterium longum* Subsp. Longum Reduces Perceived Psychological Stress in Healthy Adults: An Exploratory Clinical Trial. Nutrients.

[119] Calder P.C. (2010). Omega-3 Fatty Acids and Inflammatory Processes. Nutrients.

[120] Dimopoulou M., Savvidi S., Madesis P., Dimopoulou A., Stagos D., Gortzi O. (2026). A Review of Omega-3 Fatty Acids from Marine Source Supplements and Enhanced Food Effects on Children’s Development, Neurological and Metabolic Disorders and General Health. Mar. Drugs.

[121] Patted P.G., Masareddy R.S., Patil A.S., Kanabargi R.R., Bhat C.T. (2024). Omega-3 Fatty Acids: A Comprehensive Scientific Review of Their Sources, Functions and Health Benefits. Future J. Pharm. Sci..

[122] Li M., Li Z., Fan Y. (2025). Omega-3 Fatty Acids: Multi-Target Mechanisms and Therapeutic Applications in Neurodevelopmental Disorders and Epilepsy. Front. Nutr..

[123] Zou B., Zhao D., Zhou S., Kang J.X., Wang B. (2025). Insight into the Effects of Omega-3 Fatty Acids on Gut Microbiota: Impact of a Balanced Tissue Omega-6/Omega-3 Ratio. Front. Nutr..

[124] Hull M.A., Sun H. (2026). Omega-3 Polyunsaturated Fatty Acids and Gut Microbiota. Curr. Opin. Clin. Nutr. Metab. Care.

[125] Kumar V., Rohilla A., Ahire J.J. (2025). Omega-3 Fatty Acids and the Gut Microbiome: A New Frontier in Cardiovascular Disease Prevention. Discov. Med..

[126] Videla L.A., Valenzuela R., Del Campo A., Zúñiga-Hernández J. (2023). Omega-3 Lipid Mediators: Modulation of the M1/M2 Macrophage Phenotype and Its Protective Role in Chronic Liver Diseases. Int. J. Mol. Sci..

[127] Vomero M., Lamberti L., Corberi E., Currado D., Marino A., Berardicurti O., Fava M., Leuti A., Maccarrone M., Giacomelli R. (2025). Specialized Pro-Resolving Mediators and Autoimmunity: Recent Insights and Future Perspectives. Autoimmun. Rev..

[128] Shahinfar H., Yazdian Z., Avini N.A., Torabinasab K., Shab-Bidar S. (2025). A Systematic Review and Dose Response Meta Analysis of Omega 3 Supplementation on Cognitive Function. Sci. Rep..

[129] Mateu-Arrom L., Mora I., Garrote L. (2025). Therapeutic Benefits of Topical Omega-3 Polyunsaturated Fatty Acids in Skin Diseases and Cosmetics: An Updated Systematic Review. J. Cosmet. Dermatol..

[130] Rita El Jbeily B.S.E., Caitlyn Babi B.S., Katia Hermes B.S., Sabbaq R., Alyssa Abdelnour B.M., Sarah Shareef B.M., Kaitlin Kreuser B.M., Jessica Maloh N.D., Kurt Ashack M.D. (2024). Review of Omega-3 Fatty Acid Dietary Supplementation in Cutaneous Inflammatory Disorders. J. Integr. Dermatol..

[131] Sawada Y., Saito-Sasaki N., Nakamura M. (2021). Omega 3 Fatty Acid and Skin Diseases. Front. Immunol..

[132] Guertler A., Fiedler T., Lill D., Kuna A.-C., Volsky A., Wallmichrath J., Kämmerer T., French L.E., Reinholz M. (2024). Deficit of Omega-3 Fatty Acids in Acne Patients-A Cross-Sectional Pilot Study in a German Cohort. Life.

[133] Pu S.-Y., Huang Y.-L., Pu C.-M., Kang Y.-N., Hoang K.D., Chen K.-H., Chen C. (2023). Effects of Oral Collagen for Skin Anti-Aging: A Systematic Review and Meta-Analysis. Nutrients.

[134] Proksch E., Zdzieblik D., Oesser S. (2025). The Oral Intake of Specific Bovine-Derived Bioactive Collagen Peptides Has a Stimulatory Effect on Dermal Matrix Synthesis and Improves Various Clinical Skin Parameters. Cosmetics.

[135] Ivaskiene T., Viskelis J., Streimikyte P., Savickaitė M., Mobasheri A., Kaspute G. (2025). Collagen Supplementation and Regenerative Health: Advances in Biomarker Detection and Smart Material Integration. Front. Nutr..

[136] Gao K., Mu C.-L., Farzi A., Zhu W.-Y. (2020). Tryptophan Metabolism: A Link Between the Gut Microbiota and Brain. Adv. Nutr..

[137] Shaw C., Hess M., Weimer B.C. (2023). Microbial-Derived Tryptophan Metabolites and Their Role in Neurological Disease: Anthranilic Acid and Anthranilic Acid Derivatives. Microorganisms.

[138] Seo S.-K., Kwon B. (2023). Immune Regulation through Tryptophan Metabolism. Exp. Mol. Med..

[139] Zhou H., Pei Z., Wang N., Fang Z., Hu W., Wang H., Lu W., Zhang H., Chen W. (2026). Specific Microbiota Tryptophan Metabolites—Indole-3-Aldehyde and Indole-3-Lactic Acid Are Key Mediators in Alleviating Atopic Dermatitis. Food Biosci..

[140] Siracusa F., Machicote A., Huber S., Gagliani N. (2026). Diet-Derived Microbial Metabolites as Regulators of Immune Function. Curr. Opin. Immunol..

[141] Yoon J.-H., Do J.-S., Velankanni P., Lee C.-G., Kwon H.-K. (2023). Gut Microbial Metabolites on Host Immune Responses in Health and Disease. Immune Netw..

[142] Chen Y., Lyga J. (2014). Brain-Skin Connection: Stress, Inflammation and Skin Aging. Inflamm. Allergy Drug Targets.

[143] Bobok N., Taskesen T. (2025). Stress-Induced Changes of the Skin: A Narrative Review. Cureus.

[144] Castaño C., Novials A., Párrizas M. (2023). An Overview of Inter-Tissue and Inter-Kingdom Communication Mediated by Extracellular Vesicles in the Regulation of Mammalian Metabolism. Int. J. Mol. Sci..

[145] Dong X., Liu Y., Yang X., Li T. (2023). Extracellular Vesicle miRNAs as Key Mediators in Diet-Gut Microbiome-Host Interplay. Trends Food Sci. Technol..

[146] Viscomi B., Muniz M., Sattler S. (2025). Managing Menopausal Skin Changes: A Narrative Review of Skin Quality Changes, Their Aesthetic Impact, and the Actual Role of Hormone Replacement Therapy in Improvement. J. Cosmet. Dermatol..

[147] Sejbuk M., Siebieszuk A., Witkowska A.M. (2024). The Role of Gut Microbiome in Sleep Quality and Health: Dietary Strategies for Microbiota Support. Nutrients.

[148] Cai Y., Liu Y., Wu Z., Wang J., Zhang X. (2023). Effects of Diet and Exercise on Circadian Rhythm: Role of Gut Microbiota in Immune and Metabolic Systems. Nutrients.

[149] Suomalainen A., Nunnari J. (2024). Mitochondria at the Crossroads of Health and Disease. Cell.

